# On the Division of Tendons and Muscles

**Published:** 1842-01

**Authors:** 


### BRITISH AND FOREIGN

### MEDICAL REVIEW,

FOR JANUARY, 1842.

### PART FIRST.

### Analytical and Critical Reviews.

### Art. I.

1. *Ueber die Durchschneidung der Sehnen und Muskeln.* Von J. F. Dieffenbach. Mit 20 lith. Tafeln.—*Berlin,* 1841. 8vo, pp. 316.

*On the Division of Tendons and Muscles.* By J. F. Dieffenbach. Illustrated by 20 Lithographic Drawings.—*Berlin,* 1841.

2. *De la Ténotomie sous-cutanée, ou des Opérations qui se pratiquent pour la Guérison des Pieds-bots, du Torticollis, de la Contracture de la Main et des Doigts, des fausses ankyloses angulaires du Genou, du Strabisme, de la Myopie, du Bégaiement, etc.* Par le Docteur Ch. Phillips. Avec 12 Planches.—*Paris,* 1841. 8vo, pp. 416.

*On Subcutaneous Tenotomy, or the Operations resorted to for the Cure of Club-foot, Wry-neck, Contraction of the Hand and Fingers, false angular anchylosis of the Knee, Strabismus, Myopia, Stammering, &c.* By Dr. Ch. Phillips. With 12 Plates.—*Paris,* 1841.

I. The first of these works is from the pen of the justly celebrated professor of Berlin; and, although from the comparative lateness of its appearance, it is deprived of much of the interest with which at an earlier period it would have been received, yet as it embodies the results of this distinguished surgeon's immense experience, we shall, we are sure, perform an acceptable office in giving a somewhat lengthened notice of its contents. In a former Number (October 1839,) we analysed the works of Stromeyer, Zeis, Bouvier, and Little, and from these authors collected the main principles and practical precepts which have exercised so great an influence on the extension of the art of curing deformities. On the present occasion, therefore, we shall pass over with slight notice the opinions Professor Dieffenbach holds in common with others, and more especially occupy ourselves with those subjects in which either the skill or the boldness of the operator has suggested to him a different course. The volume is divided into twelve sections, each treating of the cure of a particular distortion, the whole being preceded by an introduction. This arrangement is convenient for the purposes of study, and will the better enable us to point out the peculiarities of the author's practice.

In the introduction, Dieffenbach yields to Stromeyer the exclusive merit of the discovery of "subcutaneous orthopædy," naming him its "inventor and founder," and awards to Guerin well-merited censure for seeking to appropriate to himself the greatest share of the credit, if not the entire discovery of the method. He justly disabuses his readers of the too prevalent idea that the simple operation of division of a tendon can alone restore a distorted limb.

"It is an erroneous conception of the nature of tenotomy to suppose it an operation which without further difficulty is capable of removing every distortion and contraction, in the manner that an incarcerated hernia is relieved by division of the strictured parts; more especially to imagine that an advanced degree of club-foot can be cured by division of the tendo achillis alone. The section of the contracted tendons does not remove the distortion of the limb, but prepares it for a well-adjusted mechanical treatment. In many contractions, on the contrary, the section of the tendon is the chief point, the mechanical after-treatment being but an incidental part of the cure, e. g. in wry-neck where the vertebræ have not become displaced, or in simple pes equinus," [the author might also have added in simple cases of angular knee contraction.] "Orthopædic operations are attended by the most brilliant success in cases where the unnatural contraction of particular muscles overpowers other muscles, the energy of which was previously normal. The result is less favorable when, in consequence of paralysis of certain muscles, their natural antagonists have so completely obtained the preponderance, that they are permanently contracted. In this case the section of the contracted tendons often induces merely an amelioration in the state of the limb; nevertheless the apparently paralytic state of the muscles is frequently removed by their restoration to activity through the cessation of the preponderance of the contracted muscles, resulting from their division. In this form of contraction, mechanical orthopædy is not simply inefficient, but aggravates the paralytic-like condition of the muscles; and the feeble limb is rendered completely unserviceable. Mechanical means are, as Stromeyer also states, equally ineffectual in spasmodic contractions. Nothing but the section of the contracted tendons and the deposition of a small intermediate tissue is capable of removing spastic contraction (habitueller krampf), precisely as in permanent contractions the normal form of the part is restored by elongation of the tendon." (p. 11.)

In the following important observations, Dieffenbach corroborates the opinions of Stromeyer:

"In the subcutaneous division of a large tendon, the achilles for example, the sheath of the tendon, contrary to what might be expected, is not divided, but in consequence of its size and laxity escapes the edge of the knife. Strempel has assured me that on the examination of subjects after death in which he had divided the tendo achillis, he invariably found one or at most two small openings in the sheath opposite to each other, and never a complete division: I have confirmed this by experiment; and the probability exists that the same result takes place in the living subject. This non-solution of continuity of the sheath is unquestionably of the utmost advantage, as it prevents extensive diffusion of blood in the adjacent cellular membrane. The promptitude with which mobility of the tendon is restored after cicatrization of the wound demonstrates that in consequence of its section, no firm adhesion of the external surface of the sheath has ensued." (p. 12.)

*Wry-Neck.* The first chapter of Dieffenbach's work treats of the nature and cure of wry-neck; but as we have already, in our notice of Stromeyer's excellent book,* furnished our readers with a summary of the history,

* Beiträge zur operativen Orthopädik. Hannover, 1838.

varieties, and treatment of this affection, we need not follow Dieffenbach through his elaborate disquisition on the subject. He disinterestedly assigns to Dupuytren the merit of having first resorted to the subcutaneous section of the sterno-mastoideus muscles, an honour which Stromeyer had erroneously awarded to Dieffenbach. The ordinary form of wry-neck arises from organic shortening of the sterno-cleido-mastoideus muscle; but, as Dieffenbach remarks, the obliquity of the neck may likewise result "from affections of the bones, ligaments, cellular tissue or skin, (cicatrices from burns;) lastly, it may proceed from an affection of the nervous system" (spastic or clonic contraction).

"If the bones be the seat of disease, its nature may be inflammatory, or a yielding of the cervical vertebræ from affection of distant parts; the deformity may also depend on paralysis of one side of the neck, and preponderating contraction of the other, principally of the sterno-mastoid. Large abscesses of long standing occasion shortening of one side of the neck, and obliquity of the head by the formation of adventitious tough cellular membrane; and still more frequently muscular rigidity (obstipitas muscularis) occurs in young children after suppuration in the neck, from the habit of inclining the head towards one side, usually towards that which is affected,—more rarely towards the opposite side." (p. 18.)

Our author's experience in this matter is founded on the successful treatment of sixty cases, which presented every variety in origin, degree, and age of the patient; the history of these is appended to the work. An interesting fact was noticed in one instance (p. 50,) namely, the occurrence of an optical delusion immediately after the operation. The obliquity of the head having been instantly removed, the patient exclaimed, "Everything appears awry, my head seems on one side," and although he looked at himself in a mirror he was not convinced of his error. This delusion continued but a short time after the operation. Troublesome hemorrhage happened in one case only (p. 51.) The patient, a healthy boy æt. 5, was operated on for congenital wry-neck, by section of the sternal and clavicular portions of the muscle of the right side. Copious hemorrhage from both punctures ensued, and the boy sunk into profound syncope. There was no jet of arterial blood, nor was any large vein wounded, the parenchymatous oozing was however as abundant as if a large vessel had been punctured or divided. After considerable difficulty and the repeated application of cold and compresses secured by adhesive plaster, the hemorrhage was arrested. The patient was on the following day able to quit his bed, the punctures united without any unfavorable symptom, and at the expiration of three weeks he was dismissed perfectly cured. In another case, (p. 52,) the professor was obliged to repeat the operation twice, before the deformity was entirely removed.

The preference to be given to the subcutaneous section is well illustrated by the success which attended his operation in a patient on whom the division of both portions of the muscle, at an interval of some weeks, by incision in the integuments, and exposure of the muscular fibres, had been previously effected without benefit. Case 60 affords an excellent illustration of the necessity of combining different surgical expedients, and advantageously shows how a master of his art like Dieffenbach can avail himself of them.

"J. S., a tailor, aged twenty, presenting scrofulous cicatrices at several articulations became affected with *caput obstipitum* in consequence of a large abscess behind the right ear which descended along the course of the sterno-cleido-mastoideus, and was accompanied with destruction of the investing integuments. A cicatrix two fingers' breadth remained and was firmly adherent to the muscle, which, together with the skin, constituted a homogeneous mass. A transverse section of the skin and muscle would have been requisite for the relief of the contraction, but I was, for well-known reasons, unwilling to swerve from the subcutaneous method. I was convinced that I could only obtain my end by two operations, performed at different periods, which I accomplished in the following manner. I dissected away the indurated ligamentous and prominent fold of skin and the adjacent condensed cellular tissue which adhered to the muscle. The portion removed was from three to four inches in length and one inch and a half in breadth. The anterior edge of the integument was then detached beneath, and by means of the twisted suture approximated to the posterior edge of the wound which constituted a fixed point. By this means the exposed muscle was invested with sound skin. Simple dressing of adhesive plaster was applied. The position of the head was as oblique as before the operation, and indeed it could not be otherwise. The most perfect adhesion had taken place on the fifth day, the last of the sutures having then been removed. Four weeks were permitted to elapse before resorting to subcutaneous division of the muscle, which I effected in the middle of its course, as notwithstanding the considerable shortening, the sternal and clavicular portions were not very prominent. The severed extremities separated fully an inch, and a considerable depression was felt at the part; the superincumbent skin was rendered tense by the retraction of the muscular fibres, and the head could be straightened. A few strips of adhesive plaster and a cravat furnished the dressing. Cicatrization ensued without suppuration, and in four weeks the head was straight." (p. 69.)

We have witnessed the successful performance of the subcutaneous operation, and are indebted to the experience of Dr. Little for corroboration of the statements of Stromeyer and Dieffenbach, that, occasionally, section of one portion only of the muscle suffices; commonly, however, the division of both is required; for although the second origin of the muscle be not prominent before the operation, it usually becomes so sooner or later after the resistance of the first is removed.

*Club-foot.* In the second section of the work we find the varieties of club-foot and their treatment elaborately discussed. The author gives a just representation of the great importance of this affection considered as a disease, and freely allows the merits of Stromeyer in originating its systematic cure.

"So long as surgery exists the name of Stromeyer will in relation to these distortions hold the first rank. The labours of his predecessors were utterly insignificant. The value of the operation for club-foot is not adequately estimated. This distortion is often a greater affliction than the loss of a limb, and many individuals formerly underwent amputation in the hope of walking better with the assistance of an artificial leg than with a club-foot." (p. 73.)

Dieffenbach himself, placed at the head of the large public hospital in Berlin, La Charité, quotes with approval the opinion of Stromeyer in favour of the establishment of hospitals exclusively for the cure of these distortions.

The frequency of club-foot is estimated ten-fold greater than hare-lip, with which it sometimes coexists. According to Dieffenbach it occurs much more frequently in boys than in girls, although the latter, he states, are the more severely affected. Boys, on the other hand, present the affection in both limbs oftener than girls. Varus and pes equinus occur in the proportion of ten of the former to one of the latter. Congenital valgus is less frequent than pes equinus. Dieffenbach estimates the absolute frequency of club-foot to be in the proportion of one to every eight hundred or every thousand births. Jörg many years since gave $\frac{1}{1000}$ as the result of his observation. Dieffenbach entertains the opinion that the left extremity is oftener affected with club-foot than the right, and that when both suffer, the left is the worse.

We have been favoured by Dr. Little with a report of the cases of *congenital* club-foot occurring in patients under twelve years of age treated at the orthopædic infirmary in this metropolis, during the first year the institution was opened: it will be seen that it corresponds in several respects with the above statements:

| | | |
|---|---|---|
| Males and females affected with congenital club-foot under the age of 12 years, | Having both feet affected | 34 |
| | ,, the right foot only affected | 23 |
| | ,, the left foot only | 6 |
| | Total | 63 |
| Males under the age of 12 years affected with congenital club-foot, | Having both feet affected | 21 |
| | ,, the right only | 13 |
| | ,, the left | 2 |
| | Total | 36 |
| Females under the age of 12 years affected with congenital club-foot, | Having both feet affected | 13 |
| | ,, the right only | 10 |
| | ,, the left | 4 |
| | Total | 27 |

All above the age of twelve have been excluded from this report; as a variety of circumstances, such as the necessity of the male obtaining his own means of livelihood, the dependence of the female upon her friends, and the greater timidity of the sex, tend, after the age of twelve, to increase in a greater ratio the number of male applicants. One cause of discrepancy between some of the results of the above list and the experience of Dieffenbach is, that our author has included in his estimate non-congenital as well as congenital distortions.

The most frequent concomitant of club-foot according to Dieffenbach is hare-lip. We have met with this combination, but according to our experience strabismus and stammering are more frequent complications. Dieffenbach has never witnessed adhesion of the fingers or supernumerary toes in conjunction with club-foot: we have observed both of these anomalies. In one instance, club-foot affected both extremities; several phalanges were absent; the perfect toes were united by a web-like membrane, and the fingers were similarly affected.

Dieffenbach recommends trial of plaster of Paris or the starch bandage in the treatment of infantile varus; although, if we may judge from his practice, he prefers the operation even in young children. Stromeyer is of opinion that a large proportion of club-feet in young children are curable without operation. We believe, however, that Dr. Little has recommended the age of from nine to fifteen months as the most favorable period for the operation, although applicable at every age. We have witnessed the practice of Dr. Little at the Orthopædic Institution, and have seen the operation no less successful at the age of two months than in a congenital case at the age of fifty-six years.

Some difference of opinion exists among authors concerning the period at which extension should be commenced, and the rapidity with which it should be conducted. The majority of orthopædic surgeons in France, Germany, and in our own country, follow the example of Stromeyer, and await the closure of the puncture in the integuments before extending the limb. Our author ranks himself among these (p. 109.) He appears however to offer an apology on behalf of those who, like Velpeau, adopt an opposite course; thus, at page 96, speaking of the violence used by Sartorius, who, in order to divide the tendo achillis, exposed it by a large incision, he says, "It should be utterly discarded in the treatment of club-foot, as the cure is attainable in a more lenient and gentle manner. It is, however, easy in the present day to censure Sartorius, but if this operator had been preceded by Stromeyer, he would unquestionably have implicitly followed the footsteps of the latter. Stromeyer justly complains, that this obsolete practice, after the general diffusion of his improved method, should have been recently reintroduced. It will at once be understood that the same objection is not applicable to the violent extension of the larger joints affected with distortion after previous *subcutaneous* division of the shortened muscles and tendons." From this quotation our readers will collect that in the treatment of club-foot Dieffenbach is an opponent of *violent* extension after subcutaneous section of muscles, whereas he does not object to the use of violence in distortions of the larger joints of the body when the same method of operating has been resorted to. A great contradiction in principle apparently exists here to which we shall have occasion to revert hereafter.

In the chapter on division of other tendons besides the achilles, in the cure of the advanced degree of club-foot (p. 101), we are surprised to observe no mention made of division of the posterior tibial. We are aware that although Stromeyer has had recourse to this operation as an important auxiliary in the treatment of the severest forms of inversion, he has not laid so much stress on this measure as we should, from our own observation, have deemed necessary. A perusal of many of Dieffenbach's cases will show the treatment to have been unusually protracted, and we are convinced that it would have been much facilitated by resorting to the same means in relation to this tendon as with regard to that of the anterior tibial and gastrocnemii muscles. Theoretically we should have arrived at this conclusion; and our conclusions, *à priori*, have been justified by the observations and practice of Dr. Little. He has demonstrated the compound nature of talipes varus, showing that it depends on a threefold alteration of the position of the foot in relation to the leg, namely, *extension*, *adduction*, and *rotation*, a condition somewhat analogous to supination of the hand. The muscles which effect this threefold alteration must be the so-called extensors (gastrocnemii) and the anterior and posterior tibial muscles with the flexors of the toes; these not only adduct the foot, but raise the inner margin from the ground. If it be true as Dr. Little states (Treatise on Club-foot and analogous Distortions, p. 226), that the law of associated actions of muscles operates pathologically as well as physiologically, we should expect the anterior and *posterior* tibial muscles to be concurrently affected with the gastrocnemii. And indeed when we examine a talipes varus, and observe the position of the foot, we cannot fail to be convinced that the posterior tibial muscle is affected as well as the anterior tibial muscle.

After determining the conjoint contraction of the muscles, the further question arises whether division of both is necessary. The object to be held in view in operating for club-foot is to remove with the knife as many obstacles to the replacement of the foot as possible: we thereby save the patient pain and expenditure of time in the after-treatment proportioned to our success in attaining the end proposed. The most perfect operation for severe club-foot, abstractedly considered, would be the division of every particle of fascia, ligament, tendinous and muscular fibre on the shortened or contracted side of the limb, which would be instantly followed by placing the limb in a straight position without the use of the slightest violence or stretching. But as this is impossible, we should endeavour to approach as nearly as possible to this desirable end, by the section of all the *actively* contracted tissues within easy reach of the knife; and among these, in many severe cases of talipes varus, is the tendon of the posterior tibial muscle. There are sundry objections to this operation in infants, which render it inapplicable; but in severe cases, especially in old subjects, it should never be dispensed with. A consideration of the bulk of the muscle, and the comparatively large size of the tendon even in an atrophied limb, will assist in the formation of an opinion on the necessity of its section. We have had the opportunity of witnessing at the Orthopædic Institution the effects of its division in a number of cases, at ages varying from twelve months to thirty-five years, and we have been informed by Dr. Little that he has never witnessed an unfavorable symptom from this operation. Its instantaneous effect in relieving the deformity is often very striking.

Dieffenbach appears to us deficient likewise in the mechanical part of the treatment. We are inclined to doubt whether, with the apparatus he describes, he has ever successfully treated the severest forms of varus (see cases 142-3-8); these we should think require a much nicer adaptation and a far more intimate application of the resources of the mechanist than those which he seems to have used. Dieffenbach is a brilliant operator and a ready inventor of the means of overcoming difficulties which beset the path of surgery; but, from a perusal of his work and from our knowledge of his character, we should think him wanting in the necessary patience and the necessary time requisite for the drudgery entailed on whoever undertakes the cure of distortions chirurgically and mechanically. Laudable and successful as have been the attempts of Stromeyer and his followers to explain to us the principles on which orthopædic treatment must be conducted, it will, on account of the frequent difficulty attending the mechanical part of the treatment, always remain to a certain extent an *exclusive* branch of practice.

The author's subdivision of the varieties or degrees of club-foot appears to us unnecessarily minute; thus he makes five degrees of pes equinus and the same of varus. Without desiring to detract from the value of this arrangement as a means of assisting the memory in relation to the severity of the cases, we should have thought that three subdivisions, at most, would have answered every purpose; and we would therefore propose the arrangement of all cases of pes equinus or varus under three heads, the ***slight, intermediate***, and ***severe***. The following remarks, in which the author treats the relative frequency of the different degrees of pes equinus, are so full of interest that we will not abridge them:

"If the prevalence of the different forms of pes equinus be examined, it will be found that the first and second grades in which the heel is moderately elevated, occur much more rarely than the fourth grade, in which the foot forms a straight line with the tibia and the individual treads on the head of the metatarsal bones. This arises either from the severer forms of pes equinus being more frequently congenital than the slighter forms, or from the slighter forms being oftener cured spontaneously, or with little assistance from art. I have observed that hare-lip, which is curable by operation, occurs much less frequently in the slighter forms, in which a small or moderate fissure in the upper lip exists, than in the severer forms in which, besides the lip, the palatal process of the superior maxillary bone, and the hard and soft palates are severed. This disproportion is somewhat modified by the circumstance that infants affected with the worst forms of hare-lip and fissured palates (Wolfs- oder Löwens-rachen) sink from atrophy produced by insufficient nutrition never becoming the subjects of operation, whereas those infants who are affected with slight hare-lip take the breast, suffer little from the deformity, and are exposed to no more risk than other new-born children. In like manner, the severe forms of pes equinus are more frequently congenital than the slighter; a portion of these also disappears, as the heel which at birth was but slightly elevated subsequently becomes depressed in the act of walking, the weight of the body operating as an extending power upon the tendo achillis and gastrocnemii. The acuteness of the angle which the foot forms with the tibia causes the entire weight of the body to act advantageously, and elongate the tendo achillis and muscles of the calf." (p. 120.)

The chapter on talipes valgus and calcaneus is a valuable contribution to our previous knowledge of the nature of these distortions. We may premise that our author employs the terms valgus and flat-foot (plattfuss) synonymously, and thus defines the characters of flat-foot:

"The flat-foot is that unnatural form of the foot in which the convexity of the dorsum pedis and the concavity of the planta pedis are changed into flat surfaces, so that the inner margin of the foot with the entire middle portion of the sole touches the ground. It is the opposite of varus in which the convexity of the instep is increased and the sole greatly hollowed out. Varus might with equal justice be named hollow-foot as valgus be called flat-foot. Club-foot arises from an unnatural contraction of the flexor muscles and rigidity of the tendons and ligaments; flat-foot, on the contrary, depends essentially on relaxation of the flexors and of the tendinous and ligamentous tissues of the foot; the ligaments which bind the tarsal bones to each other, and the aponeurosis plantaris are also relaxed. Usually a mere predisposition to flat-foot exists at birth. I have very rarely indeed witnessed the existence of the higher forms of the deformity in young children. Occasionally the valgus is aggravated by a rachitic distortion of the lower portion of the tibia and fibula. The unusual length occasionally presented in flat-foot depends partly on the removal of the arched form of the foot, and partly on the excessive separation of the tarsal bones from each other; as the resistance of the ground against the sole of the foot is distributed over the whole sole instead of being limited to the heel, the outer margin, and the fore part of the foot. No thickening of the cuticle or corns exist on the sole, but the skin is everywhere delicate and sensitive, often becoming inflamed in consequence of long standing or walking. From mere exciting causes the lower grade of flat-foot often passes on to a higher form.

"*On the exciting or occasional causes of flat-foot.* Precisely as in varus, which is on the increase, the point of the foot continually inclines more inwardly; we may observe that in valgus it becomes directed gradually more outwardly.

With the increasing growth and weight of the body, the pressure on the tarsus forces the entire foot anteriorly, owing to the yielding of the ligamentous fastenings of the individual bones. The toes are no longer pressed properly against the ground, but, as may be seen in a naked foot, during the act of walking are affected with a sort of rocking motion (schaukelnde Bewegung.) The whole of the anterior part of the foot is but little used in progression, as the patient stalks about on the posterior half: ultimately he walks on the heel only. The middle part of the sole becomes convex, the metatarsal bones and phalanges being entirely raised from the ground; the gait is rendered stiff, and resembles that of a person walking on stilts. The origin of flat-foot may be spastic, of which the following is an interesting example: (p. 227-8.)

"Case. Mr. —— aged 22, of large and powerful frame, and in robust health, became affected in early infancy with weakness of the lower extremities, which impeded walking. An examination of the limbs shows nothing abnormal either in shape or development. The feet appear perfectly well formed so long as the patient retains the sitting posture, and he can perform with facility every natural movement. But in the erect posture and in the act of walking the attitude and gait are as unsteady and tottering as those of a person walking on ice with polished soles. His gait was still more unsteady on removal of his shoes and stockings, he being then often obliged to seize hold of any object to save himself from falling. The form of the feet was natural so long as they remained simply placed on the ground; but the instant the patient rose, and the feet were thereby compelled to bear the weight of the body, they assumed the form of flat-foot. The natural hollow of the sole disappeared; the toes retracted completely, and together with the front part of the foot were raised from the ground; the dorsum pedis presenting a concave form.

"I divided in the first instance high up on the instep of the left foot the whole of the muscles which bend the ankle, the tendons flew asunder with a cracking noise, after which the front part of the foot, with the exception of the great toe which remained contracted, fell as if paralysed, and the smaller toes hung down relaxed. The contracted state of the great toe was relieved by the section of its extensor tendon. By desire of the patient I postponed the operation of the right foot until the cure of the left should be effected. The foot was retained by means of a bandage in the position it had assumed on the division of the tendons. At the expiration of a few days, neither inflammation nor suppuration having taken place, a bandage was applied from the anterior surface of the leg as low as the toes. This plan was persevered with four weeks. The limb then presented a perfectly natural appearance, and maintained its position during the act of walking. The operation was subsequently resorted to in the other foot, the extensor tendons of the fourth and fifth toes also requiring division. The after-treatment was the same, and the result equally successful; both feet perfectly resembled one another. No trace of the former condition could be detected in his gait, the soles of the feet remained hollow, and the toes were properly applied to the ground." (p. 241.)

It is evident from a careful perusal of the above extracts that the author has committed a fundamental error in the synonymous use of the terms valgus and flat-foot, being thereby led to confound not merely two entirely different varieties of deformity, but probably as many as four or five distortions differing from one another as much in external appearance as in the nature of their origin. We have seen that he defines flat-foot to be that unnatural form of the foot in which the convexity of the dorsum and concavity of the planta are converted into plane surfaces, and he mentions, as a character entirely opposed to the ordinary forms of club-foot, that the flexor muscles of the ankle (gastrocnemii and other posterior muscles of the leg), and the tendons and ligaments of the foot are in a state of relaxation. This description, as far as regards external form, is perfectly correct, if confined to true flat-foot, namely the deformity in which the individual treads unnaturally on the inner margin of the foot, the toes being more or less everted, the heel and posterior part of the sole bearing disproportionately the weight of the body, and the origin of which is relaxation of the adductor muscles of the ankle, the internal ligaments of the joint, and the plantar ligaments, muscles, and fasciæ. It is the office of the adductor muscles of the foot, and the fibrous tissues of every kind contained in the sole, to maintain the proper arch of the foot. When, however, from hereditary tendency, these structures are weaker than usual; when from imperfect nutrition of the whole system, these structures, previously of normal strength, become weakened and incompetent to resist the weight of the body; when in young people of both sexes, although well nourished, the necessity of standing or walking many hours daily, as in the occupations of errand-boy, printer, shopman, entails on the muscular and ligamentous tissues before mentioned, a greater amount of exertion and a greater stress than nature designed should occur before the attainment of the entire strength of manhood; then the abnormal form and position of the foot, correctly named flat-foot, arises. The arch of the foot, when no longer maintained by the efficient and energetic contraction of the tibial muscles, the powerful passive resistance of the deltoid and the two calcaneo-scaphoid ligaments, nor tied, as the architect would say, by the strong muscles of the sole of the foot, extending across the inferior part of the arch, and thus binding the pieces of the arch; when all these supports of one of the most elaborately-designed pieces of mechanism in the human frame are removed, the arch sinks, the astragalus is gradually depressed to the ground, and a flattened and consequently elongated everted foot remains, incapable of comfortably sustaining the weight of the body in the erect posture, and unfitted for long walks of recreation or necessity. We have heard it stated that this deformity prevails so extensively in some districts (partly, no doubt, from hereditary predisposition, but especially from poverty of diet combined with early laborious agricultural pursuits), as in Pomerania, that the government is unable to raise more than one third of the usual number of soldiers in proportion to the male population. Such is the true nature of flat-foot; but our author has included under this term: congenital talipes valgus from permanent contraction, the spasmodic t. valgus of which we have quoted from him the interesting cases given above, talipes valgus paralyticus, a deformity identical in external appearance with these, produced by complete paralysis of the adductor muscles and gastrocnemii, and lastly the rachitic form of valgus, arising, secondarily, from distortion of the knees or curvature of the tibial bones. All of these varieties of valgus must be accurately discriminated in practice, as they offer each a different prognosis and require different modes of cure. They cannot be classed with flat-foot; as besides the difference in their nature in the majority of instances, the arch of the foot is not destroyed nor the dorsum so particularly flattened as to become a diagnostic sign.

Dieffenbach has adopted a mode of operation for flat-foot similar to that we have seen resorted to at the Orthopædic Institution by Dr. Little, for the cure of the varieties of talipes valgus above enumerated. The muscles commonly contracted are the peronei, the extensors of the toes, more rarely the anterior tibial and the gastrocnemii. Often the abductors of the great and little toes and the plantar fascia require division. This fact alone will point out the difference between most of the varieties of t. valgus and flat-foot. The cases in which section of the anterior tibial is requisite, appertain more properly to t. calcaneus (hackenfuss of Dieffenbach) than to t. valgus, and those in which section of the achilles tendon is required are cases of what Dr. Little denominates talipes equino-valgus, from their partaking of the characters both of pes equinus and valgus. These subdivisions of distortions of the feet appear complicated to those who have not studied the subject; but as the varieties of deformity are so completely distinguished from each other, we must admit a correct definition and nomenclature of them can alone render the subject intelligible to those who have not made it a special study.

An operation is not necessary for the relief or cure of the slighter forms of flat-foot and t. valgus. Cessation from laborious exertion, stimulant and strengthening lotions, properly-made shoes, well-adjusted mechanical supports, according to the nature and severity of the case, often suffice for the permanent relief of the inconvenience and deformity. In those instances, on the contrary, in which the deformity has existed a considerable time, in which decided muscular contraction has taken place, and the foot cannot be restored by pressure with the hand to a proper form, in which we could not expect success by mechanical and general treatment, except after the expenditure of considerable time, to be passed perhaps in the sitting posture, an operation may be resorted to. As in many cases the surgeon has only to contend against that form of contraction of muscles which is secondary, it is of the utmost importance carefully to discriminate those really requiring division. It is extremely desirable not to weaken the foot by unnecessary division of muscles, not to consider the operation in itself as the means of restoration, but as an adjuvant to the mechanical treatment which is in flat-foot and t. valgus, of even greater importance than in club-foot; the chance of relapse after operation being greater. In talipes equinus, and in many cases of t. varus, the weight of the body, after operation, acts beneficially on the tarsus, and tends to prevent relapse, whereas in the deformities we have at present under-consideration, the greatest risk of failure arises from the difficulty of removing from the newly-restored tarsal articulations that weight they are at first so unequal to sustain.

We will now select from Dieffenbach's cases of flat-foot instances of each of the other forms of t. valgus, which we have described.

"Case. *Flat-foot. Division of the extensor tendons (flexus of the ankle) on the dorsum pedis.* Caroline S. aged twenty, a young woman of thick-set frame and scrofulous diathesis, affected with flat-foot on the right side. The dorsum pedis was flattened, the sole quite even, the malleolus internus large and prominent, the toes pointing outwards. The characters of flat-foot were rendered more evident by the act of walking, the foot then appearing more flattened.

"The patient experienced dull pains in the foot after standing any length of time. As her condition was gradually becoming more aggravated, I determined on the division of the *extensor* tendons on the back of the foot, which I effected by two punctures from without inwards. Instantly the foot fell as if paralysed, and the contracted toes assumed their natural length. I bandaged the limb and secured along the front part of the foot and leg a padded splint, by which means the foot was maintained in the position of pes equinus. The bandage was renewed after a few days; the punctures were found united; the foot continued in a straight line with the leg. A lighter form of bandage was now resorted to, and was renewed occasionally until, at the expiration of a fortnight, a common roller was the only application. Within three weeks, permission was given to make the first attempts to walk. The position of the foot was quite natural. On first placing the limb on the ground the heel did not entirely descend; a few days later the power of the foot greatly increased, and soon the gait was perfectly natural. The form of the foot was perfect, the dorsum convex, and the planta hollow." (p. 233.)

"CASE. *Flat-foot from paralysis. Division of the achilles tendon.* Paulina R. a delicate child aged twelve, was, in consequence of an attack of typhus fever in earliest infancy, affected with weakness of the lower extremity, which induced an unnatural position of the foot, and at length led to the development of a complete flat-foot. The inner margin of the foot presented downwards; the external margin upwards; the planta outwardly. The form of the foot was not much altered. The section of the tendo achillis effected the restoration of the natural position; the wound healed on the third day. The operation appeared in the first instance to have aggravated the patient's condition. In the sitting posture the foot hung powerless, and fell either inwardly or outwardly, according to the influence of gravity. In order therefore thoroughly to support the foot I applied a splint and starch bandage, by means of which the patient was enabled to walk. Exercise to any great extent was for some time prohibited, but on removal of the bandage at the expiration of a month, the foot exhibited a natural position, and the involuntary excessive mobility had disappeared. From this period the foot was daily rubbed with spirituous embrocation and methodical exercises in the manner of walking, were instituted." (p. 238.)

"CASE. *Double talipes calcaneus (Hackenfüsse); division of the flexor (extensoren) tendons on front of the ankle.* C. M., aged 4 months, was born with two flat-feet (plattfüsse) of the highest grade. The toes almost touched the tibia and the heels pointed downward. The dorsum pedis was flattened, the planta convex. I divided the whole of the flexor tendons of the ankle (extensoren) in both feet above the articulations, and after moderately depressing the feet so as to give them a natural position I applied pasteboard splints which were maintained in position by starch bandages. At every period of dressing I depressed the toes more and more until their position resembled that of pes equinus. Shortly afterwards a starch bandage was alone applied, and at the expiration of five weeks the restoration of the foot was complete, and could not be distinguished from naturally formed limbs." (p. 242.)

We shall take leave of the subject of flat-foot and proceed to one equally interesting, the cure of anchylosis and contraction of the knee-joint by subcutaneous division of the contracted flexor muscles.

*Anchylosis of the knee-joint.* Contraction and anchylosis of the knee-joint are seldom congenital affections: they are usually the result of rheumatic and scrofulous inflammation. In those produced by rheumatism, the principal seat of the rigidity is the tendinous and ligamentous apparatus of the joint; in those resulting from scrofulous disease, the state of the extremities of the bones, especially of the articular surfaces, contributes to maintain the deformity. So great, however, appears the advance of orthopædic surgery, that cases of false anchylosis of many years' standing, (we have witnessed a successful cure in which the deformity had existed twenty-six years,) which, notwithstanding former caries and participation of the heads of the bones in the mischief, have been successfully cured, that is to say, straightened with more or less perfect restoration of the movements of the joint. Dieffenbach truly remarks that "it would often seem as if *true anchylosis* had occurred as a natural means of cure, yet after the lapse of many years, the articulation admits a slight motion in the direction of flexion." It is evident from the experience of the last three years, that few incurable cases of anchylosis exist. However immoveable a joint may be on the cessation of the acute or chronic disease with which it may have been affected, the lapse of a few months or years, and the absorption of the lymph deposited in the interstices of the fibrous tissues entering into the articulation, as well as that effused between them, and which binds them to each other, leads to the reappearance of a certain mobility and prepares the limb for the skill of the orthopædist. A variety of complications at times present themselves, particularly the existence of large cicatrices in the vicinity of the joint, uniting in a mass the adjacent tendons, muscles, and bones. This source of difficulty is often easily remedied by dividing the band close to its adhesion to the bone. Another impediment is that which rests mainly on the authority of Professor Robert Froriep, the distinguished morbid anatomist of Berlin. On the dissection of an anchylosed knee, the description of which is given in his *Chirurgische Kupfertafeln,* Tab. 346, he found that even after the section of the ham-string tendons, he was prevented from straightening the knee through the resistance of the thickened fascia lata and fascia cruralis. On the separation of these fibrous bands he succeeded in extending the limb, from which he inferred that the rigidity and inextensibility of these tissues must offer a greater obstacle to the cure of deformities of the knee, than muscular contraction. A similar objection has been urged in relation to contractions of the ankle-joint, founded on the examination of a long-standing talipes dissected some time after death or after maceration. It was remarked, even after division of the gastrocnemius and other tendons, that the tarsus could not be replaced until the deltoid and other ligaments were severed. The same was observed by Mackeever.* In all these instances an oversight was committed when estimating the comparative amount of resistance offered by the muscles and other fibrous tissues after death. The greater part of the resistance of the muscles is of an active nature, and subsides soon after death, whilst that of the ligaments and fasciæ is passive, and continues until destroyed by an advanced stage of decomposition. Hence an undue importance has been attached, in post-mortem examinations, to the resistance of the non-muscular tissues. These are nevertheless a source of much difficulty, and consequently the section of contracted muscles must be accompanied, whenever practicable, by division of the shortened fasciæ and ligaments. In accordance with this principle, we have observed in the practice of the Orthopædic Institution, that for the cure of false anchylosis of the knee, Dr. Little, in addition to section of the ham-string tendons, divides whatever bands of fascia exist in the posterior part of the joint, as well as the dense prolongation of the fascia lata investing the vastus externus muscle, and adherent with this muscle to the outer condyle. We have repeatedly observed as much instantaneous relaxation of the joint result from this part of the operation, as from the separation of the muscles. The vastus externus muscle is often included in the section. Among other causes of false anchylosis of the knee, we may note severe fractures, gun-shot wounds in the vicinity of the joint, and even cicatrices of burns. These must be reckoned

* Treatise on Club-foot and analogous Distortions. By W. J. Little, M.D.—London, 1839, p. xxv.

among the least favorable cases for orthopædic operations. On the contrary, contractions that have arisen less from the extent and severity of the disease in and around the joint, than from the long retention of the limb in a state of inactivity, by which the flexor muscles have become contracted, justify the most sanguine expectations of success, and indeed, with proper management, may invariably be restored.

Our author ably states the privations often endured by those affected with these contractions, and the sacrifices they are willing to make for the obtainment of relief.

"They who have escaped from fatally destructive disease of the knee-joint with an anchylosis or contraction lead, in consequence of the inconvenient nature of the deformity, a more painful state of existence than those who have experienced a shortening of the limb from hip-joint disease. Whereas the latter are frequently enabled to walk with the assistance of a high-heeled shoe, or at the furthest with the aid of a stick, the former are frequently unable to use the limb as a support, but must submit to carry the limb suspended as a burden. The sufferers from acutely contracted knees avail themselves of an artificial leg, and many regard amputation as a blessing, to relieve them of that which has become a heavy burden to the trunk." (p. 246.)

The treatment of these contractions without operation is frequently successful. In appropriate cases frictions, manipulations, vapour and warm baths, and even the application of mechanical apparatus should be resorted to. But if much pain attend this method, irrespective of the great saving of time effected by the operation, the subcutaneous section of the contracted tissues should be preferred. The violent means which have at all times been justly condemned by the medical profession, and which in France has recently been carried to the point of extreme rashness by Louvrier (see Br. and For. M. Rev. XII. 552), should be utterly proscribed. We have often been as much amazed at the boldness of those empirics who practise this method in this country, as astonished at the patience with which it is submitted to. The presumption of the one party can only be explained, we should suppose, by the utter ignorance of medicine and surgery; and the confidence reposed by the other is attributable to the frequency with which surgeons of the first reputation declare incurable numerous cases of false anchylosis which are in reality remediable.

We have been reminded, in the perusal of Dieffenbach's work, of the circumstance, that individuals, like the entire body of a profession, may strike out boldly into a path of improvement, make the first step, usually the most important, and without a cause being assigned, stand still, leaving others, more fortunate or more skilful, to avail themselves of the information thus supplied. In our former review of works on division of tendons (October, 1839), we traced the progress of the method, and showed the long intervals during which the matter lay entirely dormant, until the brilliant genius of Stromeyer conceived the idea of the subcutaneous division of the tendo achillis, a proceeding which has since been applied by him and others to every part of the body. Dieffenbach informs us (p. 247) that nine years ago he divided the ham-string muscles for the cure of a contracted knee, the result of scrofulous caries of the joint. The case succeeded perfectly. This was subsequently to Stromeyer's discovery, a year indeed after Stromeyer had published several cases in the journals of Germany and France, although Dieffenbach may not then have noticed them, or sufficiently have appreciated them. We think it probable that the idea of dividing the ham-strings in that particular case occurred spontaneously to Dieffenbach, and yet he did not repeat the division of a tendon for the cure of a distortion, except wry-neck, until his attention (as he informs us, p. 79,) was drawn to the value of the method of Stromeyer by Dr. Little in 1836. Since that period he has operated on hundreds of different kinds of deformity. Precisely as Thilenius, Michaelis, and Delpech found no imitators, Dieffenbach derived so little confidence from his operations for the cure of a false anchylosis, as not to have followed up his first success. His inactivity in this respect is the more extraordinary, as many public writers in Germany and France were at that time actively engaged in decrying Stromeyer's operation, and we know that Dieffenbach is not the individual to be rendered inactive by the clamour or false reasonings of others.

On again resorting to division of the ham-string muscles for the cure of false anchylosis, Dieffenbach appears until very recently to have followed Stromeyer's plan of awaiting the cicatrization of the punctures, and then to have gradually extended the limb by means of the apparatus recommended by Stromeyer. In order to present to our readers the chain of circumstances and reasonings which has since led Dieffenbach to pursue a totally opposite course, and one we believe that never ought to or will be extensively adopted, certainly not by British practitioners, we shall here give the professor's own words:

"This mode of after-treatment (that of Stromeyer), the gradual extension by apparatus, was often attended with insupportable and lasting pains, and was *sometimes* opposed by unconquerable difficulties, more especially by the changes which the joint had undergone through the long duration (many years) of the deformity. I resolved therefore to institute some experiments by means of forcible straightening of the knee and other distorted articulations. The results far exceeded my anticipations, as not only the severe forms of anchylosis and contraction of the knee-joint, which could not be relieved by gradual extension, were removed by the new plan, but the time requisite for the after-treatment was reduced from years to months, and the tedious sufferings of the one method exchanged for brief but violent pain of the other.

"I shall here describe this treatment more amply, as it is applicable not only to long-standing contraction of the knee, but also to other distorted articulations. After dividing the flexor tendons of the knee by the subcutaneous method, the patient lying on his stomach and the knee projecting beyond the edge of the table, I flex the knee so powerfully as to bring the heel in contact with the buttock. This done I move the limb in the opposite direction and extend it more and more forcibly, occasionally reverting to flexion, and continue this motion of the limb up and down until it is straight. Sometimes a loud cracking is heard, arising from the tearing asunder the unnatural adhesions. I have often found the united force of three or four men necessary, particularly in adult cases, *to break a knee straight.* Even in a case of true anchylosis which had arisen from a punctured wound of the articulation and subsequent inflammation and purulent effusion within the joint, I succeeded in breaking down the union between the articular surfaces, and straightened the knee. Immediately the extension is accomplished the knee should be surrounded with compresses and a flannel bandage; the limb carefully placed in a padded tin splint, and the whole enveloped with a second flannel roller.

"This treatment appears at first view more calculated to deter than worthy of imitation. One might be disposed to warn everybody against it. It would indeed be frightful if the tendons had not been previously severed, for the violent pains which attend the method of gradual extension are produced by the tendons, even when they have been divided.

"At every removal of the bandages the limb requires to be thoroughly cleansed, and on reapplication uneven or too great pressure on any spot should be avoided, as superficial cutaneous sloughs might easily be produced. After complete cure [we presume of the inflammation and other results of the violence applied after the operation—REV.] the straightened but rigid limb should be perseveringly fomented and anointed with a relaxing unguent. The surgeon will in many cases witness the restoration of mobility in the joint, so that the individual will be enabled to walk without limping.

"In those instances of true anchylosis of the knee in the bent position which cannot be broken down, I would not shrink from undertaking to separate with chisel and saw the articulated surfaces which have grown together. I am well aware that the introduction of the point of an awl, nail, or of a knife into the healthy knee-joint has often proved fatal from suppuration and caries; I have been so unfortunate as to witness such cases, but an anchylosed joint is no longer a *joint*. This operation would hardly be more severe than that of sawing through the femur in anchylosis of the hip and knee-joints, with the view of forming an artificial articulation which has been recommended by other surgeons. The plan recently introduced in Paris by Louvrier, consisting of the sudden and violent breaking down and straightening (geradebrechung) of the knee by means of a machine, without previous section of tendons is, in my opinion, most unworthy of imitation." (p. 249.)

Have we really arrived at such a point in the history of orthopædic operations that we have to announce a retrograde movement to the unsurgical and barbarous treatment of Sartorious, a mode of treatment differing only from that above recommended by its being exercised on a smaller joint of the extremity (the ankle) instead of the larger and more important articulation of the knee? And this plan comes to us recommended by Dieffenbach one of the first surgeons of Germany! It is scarcely credible that the severe animadversions of Stromeyer on the method of Sartorius, the no less energetic remonstrance of Dr. Little in this country* against violence, and Dieffenbach's own denunciations against the employment of force in the case of distortions of the ankle, contained in the very work we have now under our notice (p. 109) should by the introduction and recommendation of "sudden forcible extension after subcutaneous division of tendons," be so completely set aside. Our author believes that the advantages of his method—sudden force after subcutaneous section of tendons—opposed to the disadvantages of the same violence applied by Louvrier by means of a machine without previous operation, arises from the simple circumstance of the operation being resorted to in the one method and not in the other. We consider the methods equally reprehensible, but if a preference could be given we believe it is not difficult to demonstrate why Louvrier's plan should be selected.

Louvrier's violence snaps tendons, tears old adhesions, fasciæ, and ligaments; violently stretches when it fails to lacerate nerves or rupture blood-vessels; yet were some or all of these results to take place, provided the skin is not torn and an external wound produced, the patient has a better chance of recovery without suppuration and gangrene than when Dieffenbach, in deference to the tendons, divides them with the

* Op. cit. p. xlix. The author cites the particulars of the method of Sartorius.

knife, leaving one or more punctures. We should sooner anticipate laceration of the skin when a puncture however small is made, as that may serve as a point from whence a rent may commence, or inflammation, suppuration, and gangrene take their origin. No increased danger can result from Louvrier undertaking to rupture tendons as well as other tissues. The rupture of the gastrocnemius tendon is comparatively a trifling accident, and if the rupture of the ham-strings was the limit of Louvrier's violence, we should not so much condemn him: but the danger in reality arises from the injury inflicted on the large nerves and vessels of the popliteal region; and if the sudden forcible *straightening* of the limb be accomplished, this injury must be equally great whether the resistance of the tendons has been previously removed by division or not.

Setting aside, for the present, the merits or demerits of Dieffenbach's plan, we are naturally led to enquire whether the premises which impelled the author to its adoption be just. We venture to assert that every case of contraction which can be immediately straightened without injury to the nerves or vessels by the "sudden forcible extension," is equally capable of restoration by the Stromeyerian method of gradual extension and without more pain; whereas, we, with equal positiveness, offer the opinion that many false anchyloses which by the "sudden forcible extension" cannot be reduced without great risk to the nerves and blood-vessels, may be restored by a more gentle process of straightening. These important structures, which after ten, fifteen, or twenty and more years' duration of the deformity have been accustomed to an altered position, have themselves become contracted or puckered up as the case may be, and sometimes bound in their new relationship to other parts by unnatural adhesions, the result of the former disease in their vicinity, may still admit the slow and gradual increase of the extending power, during which the interstitial absorption and redeposit of materials composing them necessarily accommodates them to the altered state of things. The insuperable obstacles opposed to the success of the gradual extension as asserted by Dieffenbach do not exist in cases curable without danger. Our author's experience of gradual extension is vastly different from that which we have witnessed. He speaks of the after-treatment of years' continuance being by his method reduced to months. We have seen the success of the gradual extension in cases where the deformity had existed even thirteen, sixteen, and twenty-six years, the ages of the patients varying from nineteen to forty-six years, and nevertheless the treatment in none of these cases lasted so long as twelve months. The assertion is incorrect, that after subcutaneous operation and during the subsequent gradual extension, violent pain results either in or from the divided tendons: on the contrary, the greater part of the pain experienced by this method is felt in front of the joint, about the patella, deep in the popliteal region, along the course of the nerves through the leg, and in the entire limb below the knee, owing, no doubt, to the necessary compression causing stagnation of the circulation. All these inconveniences diminish as the treatment advances owing to the numbness which ensues on continued compression of the limb.

Every one versed in the treatment of distortions after subcutaneous operation has, doubtless, discovered that after cicatrization of the punctures the progress of the after-treatment is much assisted by manipulations, that is, manual attempts to accelerate the straightening of the limb, but the *gentle violence* thus employed differs as much from the " *sudden* forcible extension" as the effects of mild doses of powerful medicines differ from the heroic doses recommended by some practitioners.

The application of statistics as a test of the relative value of different methods in medicine will, we venture to predict, favour the opponents of the "sudden forcible extension."

Of twenty-three cases treated by M. Louvrier in Paris, three suffered severe and irreparable injury. One patient died at the end of three weeks, from rupture of the nerve and other structures; the second, in whom gangrene resulted from rupture of the popliteal artery, underwent amputation; and the third sunk from sloughing within six weeks.* In none was voluntary motion or complete straightening produced. Our author relates twenty-two cases of false anchylosis of the knee, and one of true anchylosis, in which he employed " sudden forcible extension" immediately after subcutaneous section of the tendons. Recovery ensued in the majority, in periods varying from one to eight months, or longer; some suppuration in the ham occurred in several; even after complete straightening of the limb on the operating table much resistance was occasionally met with, requiring considerable compression by bandages to maintain it in position; and in two instances (cases 16 and 28) superficial sloughs and suppuration resulted from the pressure, and protracted the cure. Inflammation of the articular extremities or of the joint itself, was not a solitary event, as in case 24, in which the complete straightening of the limb was necessarily abandoned. Cases 25 and 26 terminated still more unfortunately, the one by amputation of the limb, the other fatally.

There is much reason to regret that no account of the examinations of the amputated limb nor of the post-mortem appearances in case 26 is reported by the author. It would have been interesting and useful to have been informed whether the fatal symptoms depended on injury of the vessels or nerves, and what influence the mischief to the articular tissues exercised on the result. From the rapidity of the gangrene and fatal termination of the second case, we conceive that rupture either of the nerves or blood-vessels or both had been produced. From the author's omission of the date of the operation in case 25, we cannot with any confidence determine the matter.

The only case in which any degree of sudden violence can in our opinion be justified, is that in which true anchylosis has commenced, when the adhesions between the articular surfaces, still in greater part gelatinous, begin to receive the deposit of calcareous particles. But we cannot sanction the "united exertions of three or four men" being employed to *straighten* an anchylosed limb. A jerk with the hand of the surgeon only will often enable him to detect or probably to produce mobility where none appeared to exist or really existed. The joint may thus be rendered a few degrees straighter than before the examination, and the mechanical treatment may thenceforward be conducted uninterruptedly. We would recommend the postponement of even this slight degree of violence until after the cicatrization of the punctures. We give at length, the following striking case:

* Br. and For. Med. Rev., loc. cit.

"CASE. *True anchylosis of the knee, with contraction. Section of the tendons of the semitendinosus, semimembranosus, biceps, and gastrocnemii muscles. Violent straightening of the joint. Cure.* Augustus S. æt 24, a young man of athletic frame, injured the right knee with a large sharp chisel. The wound was situated on the anterior and external part of the knee near the patella, and opened the joint. Violent inflammation of the articulation ensued, which, notwithstanding appropriate treatment by application of ice, leeches, and cooling medicines, proceeded to suppuration. The arthropyosis detached the soft tissues immediately investing the joint, and burrowed downwards to the calf of the leg. Several large incisions became necessary for the evacuations of the pus and dead cellular membrane. After long and severe suffering the wounds healed, leaving complete stiffness of the joint and contraction. A severe fall on the knee some time afterwards occasioned a fresh attack of inflammation, requiring a repetition of leeches, cold applications, &c., and the subsequent use of tepid lotio plumbi and oleaginous embrocations. The knee was bent beyond the right angle, and entirely immoveable. The heel was retracted through contraction of the gastrocnemii, the foot constituting the third grade of pes equinus. No emaciation of the leg and thigh existed, both being of equal size with the sound limb. Notwithstanding the complete rigidity of the joint, most violent pain was produced by any attempt to straighten it, or to effect further flexion of the limb. The patient moved about with difficulty, and with the assistance of crutches, and he continually sought repose in the recumbent posture. He regarded amputation as a desirable termination of his case.

"I was not prepared to expect that after section of the ham-strings I should be enabled forcibly to straighten the limb. I provided myself therefore with a species of chisel, with which I purposed to effect, if necessary, the separation of the articular surfaces. The patient was placed in the prone position on the lowest operating table, both knees projecting over the edge, and when he was firmly secured on all sides I effected the section of the semi-membranosus, semitendinosus, and biceps muscles, by two subcutaneous punctures; the operation was the more easily completed, as the leg stood perpendicularly and was held in this position by an assistant. A few drops of blood only escaped from the puncture, a somewhat larger quantity was effused beneath the skin. The patient's body, and particularly the thigh, being now fixed by several powerful assistants, three others seized the leg, and at a given signal bent it so suddenly that the heel touched the buttock, on which we immediately straightened it with the same force. A cracking noise was heard, especially when flexion was accomplished, as if dry bony matters and ligaments were torn asunder. The operation was finished by dividing the tendo achillis." [The limb was dressed as in former cases, and the patient removed home in a sedan chair.]

"The pain which had been severe at the operation, diminished within two hours afterwards, and after the escape of a considerable quantity of blood through the bandages it subsided more and more; and in a few days it remained very trifling. On dressing the limb a great quantity of partially decomposed coagula was found between the skin and bandages. The punctures suppurated slightly, that by which the tendo achillis was divided had healed by the first intention; the limb, in other respects, looked well, neither superficial nor deeper seated inflammation existing. The whole was carefully cleaned and replaced in the splint as before. Complete recovery of the patient's health took place without any general disturbance or the occurrence of a single unfavorable symptom. The limb was perfectly straight, the foot reached the ground, and the convalescent began, after the lapse of a few weeks, to walk in his apartment with the assistance of a stick. Mobility of the knee-joint began a month later, and increased from month to month, so that at present the limb is capable of considerable flexion." (p. 277.)

This must be regarded as a most *fortunate* case, but does not dispel our well-grounded fears of the violence resorted to. We entertain doubts also, notwithstanding the *cracking* and *tearing* of tissues, whether true anchylosis existed. The pain which is reported to have been produced by attempts to bend or straighten the knee before the operation indicate the existence of mobility, however slight.—One word more and we take leave of this subject. We request our readers to contemplate for a moment, apart from the danger of the method even in experienced hands, the mischievous results of recommending this method ex cathedrâ; the frightful example on the minds of the pupils of a clinical class in the sanction given to the forcible tearing asunder of a man's limb by half a dozen or more powerful assistants, more especially after a breach of surface has been effected.

*Contraction of the Hip-joint.* The cure of deformity of the hip-joint by operation was first undertaken by Stromeyer. The treatment is of course applicable to those cases only in which muscular contraction is entirely or almost entirely the cause of the lameness; cases in which dislocation or anchylosis have resulted from ordinary hip-joint disease must be excluded. In many instances, however, this disease of the joint has subsided without disorganization, the limb remaining contracted in that position in which it was held by the flexor and adductor muscles of the thigh during the continuance of the disease. The necessary frictions, manipulations, and exercise of a joint thus rendered rigid, are too often neglected, and the limb continues permanently contracted. Mechanical means may succeed in restoring mobility after the lapse of months and even of two or three years. At the expiration of this lengthened period, however, the muscles have usually become structurally shorter than natural, and a cure cannot be obtained without their division. Another class of cases, in which an operation is the sole remedy applicable, is that in which contraction of the joint has existed from birth, usually in conjunction with similar affection of the remaining articulations of the lower extremity. Here the difficulty of complete cure is greater, as the muscles are not only structurally shorter, but they are likewise spastically contracted from the diseased condition of the medulla spinalis, on which such congenital contractions depend, being usually permanent. We have been favoured with the results of subcutaneous muscular sections in cases of this kind at the Orthopædic Institution, and they justify Stromeyer's extension of his method. Our author's chapter on the subject is rich in materials for reflection. He agrees with Stromeyer as to the choice of cases, but applies his own method, "sudden forcible extension," to the hip (and elbow) as well as to the articulation of the knee. We are bound again to express our decided dissent from the proceeding in these cases likewise. All the benefit attainable from the operation in any case can be obtained by gentle mechanical means afterwards. The violent method saves trouble to the practitioner, and when successful may probably spare the patient some suffering; and, but for the risk attending it, would on that account be preferable. Careful extension and proper mechanical contrivances after cicatrization of the punctures, spare patients the suppuration and loss of skin recorded at p. 284, and the inflammation of the joint mentioned at p. 289 of our author's work.

Our author states that he has found division of the tendo achillis beneficial in cases where the patient's toe only reached the ground from the results of hip-joint disease. Unquestionably contraction of the gastrocnemius occasionally results from this cause. In every case that has come under our notice, the contraction has been slight and remediable without operation, or if more severe, and the muscle from long continuance of the contraction has become structurally shorter, no division of the tendo achillis could have been serviceable; as, in such cases, the contraction has been the necessary result of the elevation of the heel from the bent position of the knee, itself caused by the unnatural position of the hip. If the hip contraction be curable, then may division of the t. achillis be advisable. If incurable, the only effect of division of this tendon would be to force the patient to place the foot, by means of a high-soled boot, to the ground in a state of extreme flexion, a position which experience has shown us to be irksome. Patients usually prefer a boot the heel of which is higher than the toe.

We have in a former Number narrated the particulars of a highly interesting operation performed by Dieffenbach for the reduction of a dislocation of the humerus of two years' standing, by division of the pectoralis major, latissimus dorsi, teres major, and teres minor muscles. The results were worthy the Professor's fame. Two other cases are given in the work we have under our notice: in one of these the luxation had existed "many" years; reduction was not effected, in consequence probably of the glenoid cavity having become obliterated. The third case was an unreduced dislocation of the tarsus backward of one year's duration. Reduction was accomplished, but the amount of benefit derived is not mentioned.

*Contraction of the Ankle-joint.* We shall, under this head, content ourselves with the narration of one striking case:

"Case. *Old luxation of the tarsus, reduced after section of the tendo achillis.* A labourer, aged forty, fell from a considerable height. The right foot sustained the principal part of the shock, and a dislocation of the tarsus backwards was the result. The subsequent inflammation was treated by cold lotions and leeches, but reduction of the dislocation was not effected. The patient placed himself under my care a year subsequently. The length of the sole of the foot was reduced two inches, explained by an equivalent projection of the heel. The heel was also elevated the same distance from the ground. The achilles tendon was very prominent posteriorly, and with the tibia occasioned the formation of a long pyramidal interspace between them. The ankle was quite immoveable. In the act of walking the anterior part of the sole alone touched the ground, the heel remaining elevated two inches. The gait resembled that of a patient affected with pes equinus, but more laborious. On attempting to reduce the dislocation, I found the limb, as Iexpected, quite unyielding; I therefore determined on division of the tense, thick, and indurated tendo achillis. The patient was placed on a table, and the thigh and knee being securely fixed by one set of assistants, three others effected extension of the foot by means of a long hankerchief passed around it. During the gradual increase of the extension I punctured the skin on the outer side of the tendo achillis, rendered excessively tense by the extension; passed the curved point of the knife beneath the tendon, and divided it whilst withdrawing the instrument. A loud crack was heard. The assistants desisted from the extension whilst I manipulated the foot in order partially to extend the ligaments and facilitate the restoration. The extension was recommenced with increased vigour, during which I, with considerable force, reduced the luxation. The foot and leg were then enveloped with bandages, saturated with solution of starch, and the patient was removed to bed. The symptoms which followed were quite unimportant, and in a few weeks' time the patient was discharged from my care." (p. 302.)

*Contracted fingers and toes.* Under the head of treatment of contracted fingers and toes by operation and "forcible extension," among several cases successfully treated by this method, is one (case 58) which bears us out in our condemnation of the method when applied to the larger joints, as the violent extension at the time of the operation did not succeed in straightening the finger, whereas gradual extension afterwards succeeded perfectly.

*Curvature of the spine.* Dieffenbach does not speak favorably of the division of the muscles of the back; and as his experience must have great weight, we will here quote his very impartial opinion. We must premise, however, that Guerin unjustly receives the merit of having first proposed the section of spinal muscles. Stromeyer divided the rhombodei and latissimus dorsi for a lateral curvature before 1834. This reclamation, due to Stromeyer has, as far as we know, never before been publicly made. He did not then extend the operation, entertaining no confidence in its results.

"Guerin's operation of dividing contracted muscles of the back for the cure of spinal distortion has excited great wonder. In the first place an important objection may be offered, that slight distortions induced by augmented muscular action are curable without operation, whereas in severe deformity of the spine, little benefit can be expected from section of muscles. These objections can, however, have no weight if experience decide favorably on the value of Guerin's method. I have once only tried it in scoliosis, and witnessed no more advantage than I had anticipated, namely, some trifling amendment." (p. 312.)

*Muscular spasm.* A more interesting contribution to surgical science is the treatment of a case of chronic twitching of the muscles of the face by subcutaneous tenotomy. The case is unique in the annals of orthopædy, and does as much honour to Dieffenbach for his skill in its performance as is due, to use our author's words, to "the talented Stromeyer who first proposed it." We will conclude our notice of Dieffenbach's volume with an abridged translation of it.

"CASE. *Chronic spasm of the muscles of the face; subcutaneous division of the muscles.* Herr U., æt. 43. Nine years previously he was suddenly seized with a spasm of the *orbicularis* of the right eye, in consequence of exposure to a draught of cold air when heated. The convulsive spasms gradually extended over the whole of the muscles of this side from the forehead to the chin, accompanied by severe headach and lacrymation. The face was constantly drawn into the strangest grimaces; the forehead wrinkled, the eyelids alternately opening and shuting, and the corner of the mouth drawn forcibly upwards by the *zygomatici* and *levator anguli oris*, so as to prevent or greatly impede speaking. He could temporarily relieve these distressing spasmodic actions by strong pressure with his hand on the cheek, so as to steady the muscles. On the other hand, the voluntary contractions of the *orbicularis* in the attempt to shut the eye would induce the spasms; and hence he had difficulty in going to sleep on account of the spasm excited on closing the eyes. The operation was performed on the 11th June.

"A long pointed fistula-knife was inserted under the skin of the right corner of the mouth and thrust upwards under the skin to the lower border of the under eyelid. The cutting edge of the knife was then turned towards the muscles, which were divided, in retracting the knife, without touching the skin. By this means the lower portion of the *orbicularis*, the *zygomatici*, and the *levator ang. oris* in their middle, were divided. The knife was then introduced through the mucous covering of the outer angle of the eye, passed on under the skin, and once more divided the *orbicularis* on its retraction. A third puncture was made upwards through the mucous portion of the upper lip beneath the nose, and the *depressor alæ nasi* divided. The morbid muscular twitchings were seen to diminish after every incision. A firm compress of charpie and sticking-plaster was laid over the whole side of the face, and cold poultices applied for several days. After this the ecchymosis and swelling gradually subsided, and the cheek assumed its healthy aspect. At first some feeble twitches were observed, which after changing to a slight vibratory movement of the inner angle of the eye, finally disappeared." (pp. 315-16.)

II. The work of M. Phillips, as its title expresses, does not relate solely to distortions of the limbs, but includes the treatment of squinting and stammering. We are inclined to believe that by this time the present knowledge of these subjects is nearly exhausted; at all events we doubt not that the patience of the readers of our numerous journals is nearly so. We shall therefore confine our notice of M. Phillips's book to those parts which refer to distortions of the limbs and wry-neck. Much of this author's experience appears to have been obtained in Berlin. He is a close follower of Dieffenbach, to whom indeed the work is dedicated; and it has been published so quickly after the treatise we have already noticed as not to contain much that is novel or important. Our task therefore will chiefly consist in selecting whatever of the latter we can find, and in recording those opinions and points of practice in which he differs from the best authorities.

One of our offices as reviewers—and by no means an agreeable one—is to set the professional public right as to the origination of curative methods or theories. Such an appropriation would be unimportant beyond satisfying a sense of justice, were it not for the circumstance that the value the public is disposed to set upon a theory is determined and enhanced by the knowledge of the literary and professional character of the propounder. We do not wish to imply that these observations are specially called for in relation to our present subject: on the contrary, they apply generally. No subject in medicine or surgery ever made such rapid strides as that of subcutaneous section of muscles and tendons during the short period that has elapsed since what we may almost term its discovery by Stromeyer in 1830. The extension of the method from wry-neck and talipes to every articulation of the extremities, and to other organs, as the eye and tongue, has been so rapid, and the modifications in the mode of operating and the kind of instruments and apparatus employed have been so numerous, and perhaps often simultaneously adopted by practitioners in different countries, that when perusing the treatises of various authors we have been continually perplexed at meeting with the most opposite assertions as to the claims of priority. This is in our opinion accounted for mostly by the above explanation, and partly from successive authors having been imperfectly acquainted with the published labours of their predecessors. A comparison of dates of the first operations of different authors shows that Stromeyer found imitators in France, in the persons of Bouvier, Duval, Cazenave, and Guerin, and among our own countrymen in the persons of Whipple and Little, before the method was embraced in its native country by Dieffenbach. It is true that its adoption was nearly simultaneous by many, yet the inactivity of Stromeyer's countrymen is remarkable, and illustrates the old proverb of the prophet's want of honour in his own land. The merit of the first performance of the operation for strabismus is due, we believe, to Dr. Pauli of Dresden, although Stromeyer had previously recommended it. The credit of the suggestion has been unjustly claimed by Guerin. We have elsewhere mentioned that Stromeyer long since entertained and acted upon the idea of curing spinal distortions by muscular sections. The priority in this matter has likewise been assumed by Guerin. The charitable supposition is that Guerin may have been so incessantly whirled in the sphere of his own orthopædic practice that he has directed little attention to the examination whether the ideas he has emitted have originated with himself or with others. Hence almost every theory and point of practice in orthopædy has been claimed by him. These reflections have been suggested by the perusal of the treatise of M. Phillips. He is exceedingly severe against Guerin; we are ourselves disposed to coincide in the propriety of many of his censures, although we are reluctantly compelled to believe that pure love of science is not the only source of them. So much acrimony lurks throughout his observations that we fear professional jealousy has had no small share in their production. In M. Phillips's catalogue of complaints against Guerin the claim to the discovery of the etiology of congenital distortions might have been included. Stromeyer, in his little work formerly noticed by us "Ueber Paralyse der Inspirations-muskeln," laid the basis of the theory of their dependence on muscular contraction, afterwards (in 1836) fully demonstrated by Dr. Little, from his own researches and from the testimony of B. Bell, Duverney, Boyer, Jörg, Rudolphi, and others. Guerin, notwithstanding, appropriates to himself the origination of the theory.

A curious explanation of the difference in the etiology of talipes equinus and t. varus is offered by M. Phillips, viz. that contraction of the internal part of the gastrocnemius, or probably of the soleus, only produces t. varus; whereas contraction of the whole of the muscles of the calf induces t. equinus. Such might be the difference in the cause of these varieties of distortion if the origin of the contraction were mechanical; but, as Dr. Little has proved, these distortions have a dynamic origin. The circumstance that determines the production of t. equinus or t. varus, is the greater or smaller number of filaments of nerves, affected at their origins in the medulla spinalis. If those distributed to the extensors of the leg be excited, spasmodic contraction of the muscles supplied by them will be induced, and the spastic form of t. equinus result; but if the nerves supplying the adductor muscles, as well as those of the extensors be involved, t. varus will ensue. *External* causes may modify or augment the deformity, but the distinctive characters of the individual forms are as well defined as those of variola and rubeola. We think M. Phillips too warmly disposed towards the operation. He is certainly much in error when, in treating of the possibility of cure of club-foot by mechanical means, he says, p. 44, "It would be superfluous to resort to them for the cure of congenital club-foot; the power of the muscles would inevitably prove too great for the successful use of apparatus." We deduce from this observation either that our author has never attempted the cure of congenital club-foot without operation, or that having attempted it, he has failed through the selection of improper cases, or from the employment of inadequate mechanical contrivances. Instances of cure must have occurred occasionally within the experience of most surgeons prior to the successful introduction of the method by operation. We have recently witnessed cures effected by the mechanical means, it being the practice at the Orthopædic Institution to treat without operation all infantile cases not of the higher grades of severity.

The following remarks on redivision of tendons suggest grave matter for reflection:

"The repetition of the operation is more especially useful in talipes equinus of the third degree, or in severe t. varus when inversion of the foot exists. This second operation should never be performed at the same situation as the first, as sufficient power of reproduction does not exist: the vitality of the part is diminished, and the capability of reunion insufficient. The risk of the divided ends remaining separated, ununited, would be incurred. The redivision should consequently take place above the part first divided: the newly-cut surfaces receives its blood-vessels in a more direct manner, and reunion is as perfect as at the first section. It is usually requisite to divide the tendo achillis several times, for the completion of the cure of severe talipes equinus. A young man affected with the highest degree of t. equinus, underwent at Berlin the division of the tendo achillis twenty-two times. I was present at the last four operations, which were done within the period of a few weeks." (p. 54.)

We regret to be obliged to dissent from almost every statement contained in this extract. We have never found redivision of the tendo achillis necessary in any, even in the severest instances of t. equinus; and with respect to the highest grade of t. varus, we have but rarely effected the section three times. The cases of t. varus, in which we have been compelled to repeat the operation, have been in those of adults affected with severer distortion than any figured in either of the works before us represent.

Mr. Phillips's opinion of the point at which redivision should be effected is derived from the practice of Dieffenbach, and rests upon a fundamental physiological error. He assumes that the arteries of the lower portion of the tendon are derived entirely from the upper portion, and consequently that after the first section and subsequent healing, the function of the arteries supposed to supply the lower portion must be arrested or diminished, so that the operator on redividing the tendon below the first divided, would be exposed to the chance of witnessing continued separation and non-union of the divided extremities. It is contrary to fact to suppose that the tendo achillis, for instance, is not supplied with blood from below and from the adjacent cellular tissue: were it not so, the lower extremity of the tendon would be as incapable of performing its share of the restorative process, by the effusion of lymph or extension of its vessels, or by granulation, after the first section as after the second. The experiments of Von Ammon, Gunther, and Bouvier have demonstrated that the organization of the effused lymph or coagulum formed between the severed extremities of the tendo achillis proceeds equally from both. We may also remark, that even if it could be proved that tendons receive their supply of blood solely from above downwards, it would not serve as an argument against the possibility or probability of union taking place in the part divided below a former section. For a certain period after union had ensued in the part first operated, the quantity of blood circulating through the uniting medium would be greater than through an equal portion of original tendon, the organization of the

4290

intervening lymph being greater than sound tendon, and it has been demonstrated that the adjacent parts of the tendon continue for a time swollen and the vessels larger. Experience also proves that when redivision of the tendo achillis is performed below the part first operated, perfect reunion ensues. We have been informed that it is the invariable practice of Dr. Little, when a second operation is required, to sever the tendon below the part first incised, and the reasons he assigns for this preference are that in the severe forms of t. varus, in which alone it should be required, the tendon is placed at so little distance from the deeper tissues, that it becomes often adherent to their investing fascia; consequently, the performance of the second operation above such adhesion might impede the descent of the heel, whereas its performance below the point first operated on readily permits the necessary elongation of the part. The surgeon who is about to operate on so severe a form of t. varus, that the necessity of again resorting to the knife appears not unlikely to occur, should take a hint from this reasoning and experience, and divide the tendon high up instead of near to the os calcis.

We must more especially protest against the practice which, if not positively recommended by our author as worthy of imitation, is tolerated by him as necessary, namely, the division of the tendo achillis on twenty-two successive occasions, in the same patient. We confidently aver, unless the case were from some peculiar obstacle incurable, which it does not appear to have been, that if the same amount of time and pains had been properly bestowed on twenty-two occasions to the mechanical after-treatment of the patient, the repeated operations might have been dispensed with. The same censure awaits the practice M. Phillips (p. 80) quotes from Dieffenbach, that of being obliged to divide the tendo achillis four times before commencing the mechanical treatment. This early redivision must be totally unnecessary, unless the surgeon has neglected to prepare his mechanical apparatus beforehand.

The chapter of M. Phillips devoted to the consideration of t. valgus merits perusal, as it is a more accurate definition of the varieties of this deformity than any we have met with.

The chapter on the cure of congenital and acquired contractions of the hand, although not containing much original matter, raises some very important questions. Much more extended experience of the utility of sections of numerous muscles of the hand and arm is necessary before we can lay down any precise rules by which the practitioner can be guided either in the selection of cases, or in the mode of operation. In principle, subcutaneous orthopædy is equally applicable to the upper and lower extremities, but a wide difference exists in the application of the principle. The movements of the ankle consist, it is true, of extension, flexion, adduction, and abduction; but in the act of walking, flexion and extension are alone absolutely necessary; adduction aud abduction of the foot are only required under extraordinary circumstances. Surgical orthopædy is capable of restoring a useless leg, and fitting it for the ordinary purposes to which in the civilized state of society it is applied; but cannot adapt it to the extraordinary use made of it by the athletes or professional dancer. But in the case of the hand, every individual from the day labourer to the watchmaker, or the workman in the minutest branch of the arts, avails himself constantly not only of flexion and extension, but of pronation and supination, those movements which are analogous to the inversion and eversion of the foot. If we at the same time bear in mind that, notwithstanding the analogy in these movements of the upper and lower extremities, the acts of pronation and supination are far more delicate and elaborate than the analogous movements of the foot, if we remember that not only the movements of the hand are much more complicated, but that the several fingers possess each their allotted muscles and consequent functions, we shall at once perceive that although in principle orthopædic operations are equally applicable to the hands, the difficulty of applying the method must be immeasurably greater. The anatomical conformation of the arm and hand, the smaller space in which is congregated a larger number of nerves, muscles, and tendons, the difficulty of dividing those which may be contracted without disturbing or exciting unnatural adhesion with those which may be normal in their action,—all combine to retard the progress of orthopædy in this direction. We have already remarked when speaking of Dieffenbach's views on this subject, that operations of the elbow are usually successful; in the hand also when merely unnatural flexion or extension exists, whether the result of congenital contraction, or acquired from dental irritation, from long continuance of the wrist in either of these positions, or the result of local inflammations, abscesses without great loss of substance, and sprains and fractures,—the functions of the wrist may be restored. Thus we have witnessed at the Orthopædic Institution cases of contracted elbow and wrist from all of these sources which have yielded perfectly by section of the biceps, or of the flexors and extensors of the wrist, the functions having been completely restored. We have also seen division of the supinators and pronators followed by more or less complete restoration of the deficient functions, according to the cause of the contraction. We may briefly state the order of success in these cases to have been as follows: organic shortening of muscles from long-continued relaxation, as from accidental injuries and inflammations; congenital contractions; spasmodic contractions from fever or other general disturbance during infancy; contractions from paralysis of antagonists, and contractions from abscesses with loss of substance, grangrene, &c. The result of our observation leads us to assert that, however discouraging at the outset, contractions of the hand are not irremediable; on the contrary, although few are perfectly curable, the great majority are susceptible of considerable improvement. Although the degree of restoration of function varies, yet in nearly every case that has come under our notice, the deformity which is alone sufficient to impel individuals of the upper classes of society to seek relief, has been removed. A wide field however remains open for future research and experience; and although we would strenuously discountenace all rash and wholesale division of tendons in these cases, we venture to recommend the matter to the attention of those who are possessed of an intimate knowledge of the anatomy of the parts, and are endowed with a large share of patience to watch and elaborate results. The loss of a lower extremity is a great privation, but experience has convinced us that the deprivation of the use of an arm and hand is felt as a far greater affliction; so much the greater therefore must be the reward of him, who by adding to the common stock knowledge on this subject, can so largely contribute to the welfare of his fellow-creatures.

M. Phillips has devoted a chapter to the cure of false anchylosis of the knee. He advocates the violent extension of Dieffenbach, the impropriety of which we have already sufficiently discussed, and need not therefore again refer to it. We perceive however that M. Phillips has not encountered the fatal difficulties described in some of Dieffenbach's cases, obviously because he has wanted the boldness (rashness?) to follow out Dieffenbach's proceeding to the utmost; he was consequently compelled to trust much more to the slower mechanical after-treatment. The cases of false anchylosis, related by M. Phillips, do not impress us very favorably with the results of the operation in his hands.

Our notice of these works must now be brought to a conclusion. We have endeavoured to acquaint our readers with the more interesting of the contents; but we beg at the same time to recommend both for perusal, more especially Dieffenbach's volume, which possesses such abundance of materials. We may be considered somewhat severe in our strictures; but when it is considered how much and irreparable injury may be inflicted on a chirurgical method still new, by the incautious, unreflecting manner in which orthopædic operations are resorted to, we think, notwithstanding the European reputation of Dieffenbach as a surgeon, we ought not to hesitate to state what we regard as his errors, and wherein we differ from him. His book loses much of its utility from the hasty manner in which it has been written. The numerous cases which might have been rendered of immense importance to the statistics of deformities, are drawn up without dates or mention of the precise results derived after a given period of treatment. Thus we are often told that the deformity had existed "many years," "from the earliest period;" that at the expiration of "some weeks," "a few months," the patient was restored, &c. And we miss those minutiæ of anatomical relation, that exact description of the pathological condition, without the possession of which the practitioner cannot compare results and judge for himself of the value of different methods of treatment.

---

